# Unraveling the PANoptosis Landscape in Osteosarcoma: A Single-Cell Sequencing and Machine Learning Approach to Prognostic Modeling and Tumor Microenvironment Analysis

**DOI:** 10.1155/ijog/6915258

**Published:** 2025-03-20

**Authors:** Xue-yang Gui, Jun-fei Wang, Yi Zhang, Zi-yang Tang, Ze-zhang Zhu

**Affiliations:** ^1^Department of Orthopedic Surgery, Nanjing Drum Tower Hospital, The Affiliated Hospital of Medical School, Nanjing University, Nanjing, China; ^2^Department of Orthopedic Surgery, Nanjing Drum Tower Hospital, The Clinical College of Nanjing Medical University, Nanjing, China

**Keywords:** bioinformatics, cell death, osteosarcoma, PANoptosis, scRNA

## Abstract

**Background:** Osteosarcoma (OS) is a highly aggressive bone malignancy prevalent in children and adolescents, characterized by poor prognosis and limited therapeutic options. The tumor microenvironment (TME) and cell death mechanisms such as PANoptosis—comprising pyroptosis, apoptosis, and necroptosis—play critical roles in tumor progression and immune evasion. This study is aimed at exploring the PANoptosis landscape in OS using single-cell RNA sequencing (scRNA-seq) and at developing a robust prognostic model using machine learning algorithms.

**Methods:** Single-cell sequencing data for OS were obtained from the GEO database (GSE162454), and bulk transcriptome data were sourced from the TARGET and GEO databases. Data integration, dimensionality reduction, and cell clustering were performed using UMAP and t-SNE. PANoptosis-related genes were identified, and their expression profiles were used to score and categorize cells into PANoptosis-high and PANoptosis-low groups. A comprehensive prognostic model was constructed using 101 machine learning algorithms, including CoxBoost, to predict patient outcomes. The model's performance was validated across multiple cohorts, and its association with the mutation landscape and TME was evaluated.

**Results:** The scRNA-seq analysis revealed 14 distinct cell clusters within OS, with significant PANoptosis activation observed in cancer-associated fibroblasts (CAFs), myeloid cells, osteoblasts, and osteoclasts. Differentially expressed genes between PANoptosis-high and PANoptosis-low groups were identified, and cell communication analysis showed enhanced interaction patterns in the PANoptosis-high group. The CoxBoost model, selected from 101 machine learning algorithms, exhibited stable prognostic performance across the TARGET and GEO cohorts, effectively stratifying patients into high-risk and low-risk groups. The high-risk group displayed worse survival outcomes, higher mutation frequencies, and distinct immune infiltration patterns, correlating with poorer prognosis and increased tumor purity.

**Conclusion:** This study provides novel insights into the PANoptosis landscape in OS and presents a validated prognostic model for risk stratification. The integration of scRNA-seq data with machine learning approaches enhances our understanding of OS heterogeneity and its impact on patient prognosis, offering potential avenues for targeted therapeutic strategies. Further validation in clinical settings is warranted to confirm the model's utility in guiding personalized treatment for OS patients.

## 1. Introduction

Osteosarcoma (OS) is the most common primary malignant bone tumor in children and adolescents, characterized by high aggressiveness and poor prognosis [1–3]. Despite advances in multimodal therapies, including surgery, chemotherapy, and radiotherapy, the survival rate for OS patients has not significantly improved over the past few decades [4–6]. The complex and heterogeneous nature of OS, coupled with its intricate tumor microenvironment (TME), contributes to therapeutic resistance and metastasis, underscoring the need for novel prognostic models and therapeutic strategies [7–9].

Recent advances in single-cell RNA sequencing (scRNA-seq) have provided unprecedented insights into the cellular heterogeneity and dynamic states within tumors, enabling the identification of distinct cell populations and their interactions within the TME [10–12]. Among the various cell death mechanisms, PANoptosis—a unique inflammatory cell death pathway encompassing pyroptosis, apoptosis, and necroptosis—has emerged as a crucial process in cancer biology, contributing to tumor progression, immune evasion, and therapeutic resistance [13–16].

This study is aimed at elucidating the PANoptosis landscape in OS using scRNA-seq data and at constructing a robust prognostic model by integrating PANoptosis-related gene expression profiles with bulk transcriptome data from multiple cohorts. By leveraging advanced machine learning algorithms, we sought to develop a predictive model that can stratify patients based on their risk of poor outcomes, providing valuable insights into the underlying mechanisms of OS progression and potential therapeutic targets.

## 2. Materials and Methods

### 2.1. Data Download

Single-cell sequencing data for OS were sourced from the GEO database (GSE162454), comprising tissue samples from six patients diagnosed via surgical resection [16–18]. Quality control criteria included the following: (1) nFeature_RNA between 200 and 7000; (2) mitochondrial genes < 20%; (3) hemoglobin genes < 10%; and (4) nCount_RNA < 100,000.

Bulk transcriptome data for OS were obtained from the TARGET and GEO databases. Initially, sequencing and survival data for the OS patient cohort were downloaded from the TARGET database. Subsequently, gene expression matrices and survival data for the OS cohorts GSE16091 and GSE21257 were acquired from the GEO database [19, 20]. The two datasets were combined into a GEO superset for validation purposes. Batch effects arising from different platforms were mitigated using the R package “sva”[21].

### 2.2. Construction of OS Single-Cell Sequencing Landscape and Exploration of PANoptosis Heterogeneity

Highly variable genes were normalized, and the Harmony method was used to integrate the data and remove batch effects. Dimensionality reduction was performed using UMAP and t-SNE methods. Cell clusters were annotated based on biomarkers for known cell types. Cells were then scored for enrichment of PANoptosis-related genes using the “AUCell_calcAUC” function from the AUCell package [22]. Cells were divided into high and low groups based on the median score, and differentially expressed genes between the two groups were identified. The “CellChat” R package was used to analyze differences in cell communication and interactions between the two groups. Genes highly correlated with PANoptosis scores were identified using the “cor.test” function, and these genes were intersected with the differentially expressed genes to identify those most relevant to PANoptosis.

### 2.3. Construction of the Optimal Prognostic Model Using 101 Machine Learning Algorithms

The gene set was first subjected to COX regression to identify genes significantly associated with prognosis. Then, 101 machine learning algorithms and their combinations were employed to construct prognostic models, and their *C*-index was calculated [23]. These algorithms included StepCox, Lasso, Ridge, partial least squares regression (plsRcox), CoxBoost, random survival forest (RSF), generalized boosted regression modeling (GBM), elastic net (Enet), supervised principal components (SuperPCs), and survival support vector machine (survival-SVM). The models were ranked based on their *C*-index, and the highest-ranking model was selected as the optimal prognostic model.

### 2.4. Evaluation of the Prognostic Model's Value

Risk scores for patients were calculated based on the prognostic model in the bulk transcriptome datasets, including TARGET, GSE16091, GSE21257, and the GEO superset. Patients were divided into high-risk and low-risk groups based on the median risk score. Survival curves for different cohorts were plotted using the “survival” and “survminer” R packages to evaluate the model's prognostic value. ROC curves were constructed for the four cohorts using the “timeROC” R package, and the area under the curve (AUC) was calculated to assess the model's accuracy. Finally, PCA curves for different cohorts were constructed using the “PCA” function to explore the model's ability to distinguish between high-risk and low-risk patients.

### 2.5. Mutation Analysis

Mutation binary matrices for OS were downloaded and converted into 0–1 matrices. Tumor mutation burden (TMB) data were downloaded, with outliers removed and data standardized. Copy number variation results were retrieved from the GISTIC2.0 database to identify amplified and deleted sites. Mutation data and risk files were merged, and a heat map was used to display the mutation landscape, calculating differences in TMB, amplifications, and deletions between high-risk and low-risk groups.

### 2.6. TME Analysis

Immune microenvironment analysis was conducted by downloading immune cell infiltration data for OS, merging it with risk stratification data, and using Kruskal–Wallis and Wilcoxon tests to calculate differences in immune cell abundance, visualized with a heat map. TME scoring was performed by downloading TME-related data from TCGA database, merging it with risk stratification data, and removing outliers. Correlations between risk scores and various TME scores were calculated using the “rcorr” function. Gene set enrichment analysis (GSEA) was used to identify differentially enriched signaling pathways between high-risk and low-risk groups.

### 2.7. Statistical Analysis

Statistical analyses were performed using R version 4.3.2. For differential gene expression analysis, the “limma” package was used, and the threshold for statistical significance was set at a *p* value of < 0.05. The prognostic model was constructed using 101 machine learning and ensemble algorithms, with model performance evaluated based on the concordance index (*C*-index) and area under the receiver operating characteristic curve (AUC). Batch effects were corrected using the “sva” package's comBat function. The correlation between gene expression and survival outcomes was assessed using the Cox proportional hazards regression model. Statistical significance was determined with a log-rank test for survival analysis.

## 3. Results

### 3.1. Single-Cell Sequencing Analysis Reveals the PANoptosis Landscape in OS

**Figure 1:**
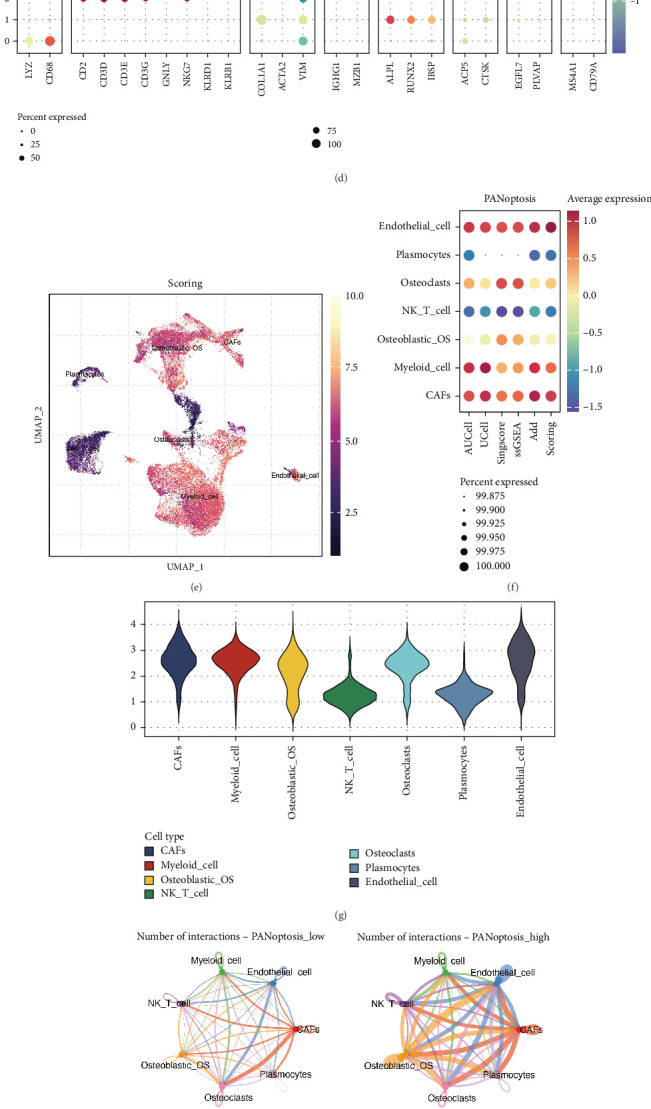
PANoptosis landscape in osteosarcoma identified by single-cell sequencing analysis. (a) UMAP plot showing the distribution of 14 distinct cell clusters identified from osteosarcoma single-cell sequencing data. (b) Annotation of cell types within the clusters, including cancer-associated fibroblasts (CAFs), myeloid cells, osteoblasts, NK/T cells, osteoclasts, plasmocytes, and endothelial cells. (c) t-SNE plot illustrating the separation of annotated cell types. (d) Heat map showing the expression of key marker genes used for cell type annotation. (e) PANoptosis enrichment scores across different cell types, highlighting significant activation in CAFs, myeloid cells, osteoblasts, osteoclasts, and endothelial cells. (f) Violin plot displaying PANoptosis scores for the cell types with significant activation. (g) UMAP plot showing the distribution of PANoptosis-high and PANoptosis-low cells based on median PANoptosis score. (h) Cell communication analysis revealing distinct communication patterns between PANoptosis-high and PANoptosis-low groups. (i) Heat map of the communication strength between different cell types in PANoptosis-high and PANoptosis-low groups. (j) Detailed analysis of incoming and outgoing communication patterns for NK/T cells and CAFs in both PANoptosis-high and PANoptosis-low groups. (k) Correlation analysis identifying genes most associated with PANoptosis scores, with significant genes used for subsequent prognostic model construction.

First, we identified that single-cell sequencing data from OS can be classified into 14 clusters (Figure 1a), which were annotated as cancer-associated fibroblasts (CAFs), myeloid cells, osteoblasts, NK/T cells, osteoclasts, plasmocytes, endothelial cells, among others (Figures 1b,c). The annotations and markers used are shown in Figure 1d. Using PANoptosis-related genes, we performed enrichment scoring on these cells and found significant activation of PANoptosis in CAFs, myeloid cells, osteoblasts, osteoclasts, and endothelial cells (Figures 1e, 1f, and 1g). Based on the median PANoptosis score, all cells were divided into PANoptosis-high and PANoptosis-low groups, and differentially expressed genes between the two groups were identified. Cell communication analysis revealed more frequent communication patterns in the PANoptosis-high group (Figures 1h, 1i, and 1j). Notably, NK/T cells exhibited a strong incoming communication pattern in both groups, while CAFs demonstrated a strong outgoing communication pattern (Figure 1j). Additionally, correlation analysis identified genes most associated with the PANoptosis score (Figure 1k). These were intersected with the differentially expressed genes to construct a prognostic model.

### 3.2. Prognostic Model Construction for OS Using 101 Machine Learning Algorithms

**Figure 2:**
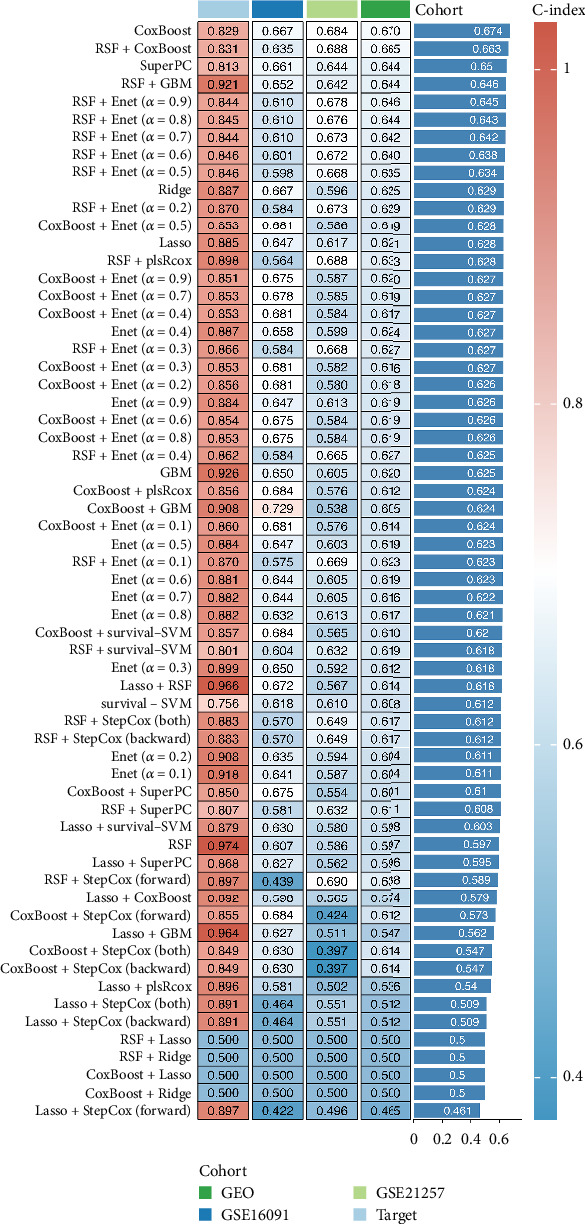
Construction of prognostic model for osteosarcoma using 101 machine learning algorithms: The CoxBoost model was selected as the optimal model based on its consistent performance across all cohorts.

After identifying the modeling genes, we constructed a prognostic model using a combination of 101 machine learning algorithms. The results indicated that the CoxBoost model exhibited the most stable performance across the TARGET cohort, GSE16091 cohort, GSE21257 cohort, and GEO-total cohort and was therefore selected for subsequent analysis (Figure 2).

### 3.3. Clinical Validation of the CoxBoost Prognostic Model

**Figure 3:**
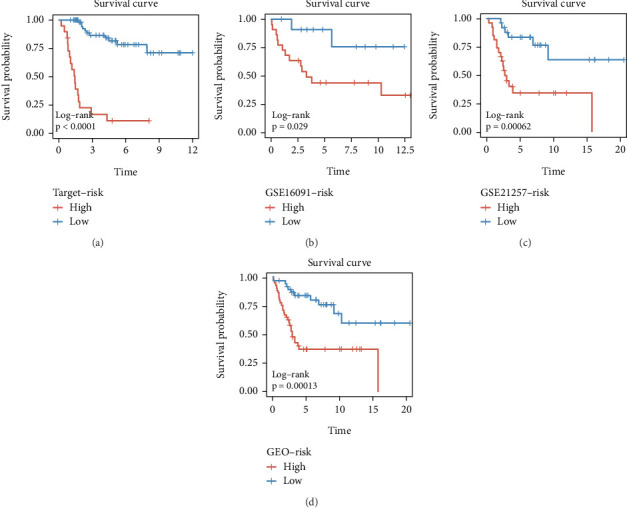
Clinical validation of the CoxBoost prognostic model. (a–d) Kaplan–Meier survival curves comparing high-risk and low-risk groups within the (a) TARGET cohort, (b) GSE16091 cohort, (c) GSE21257 cohort, and (d) GEO-total cohort.

**Figure 4:**
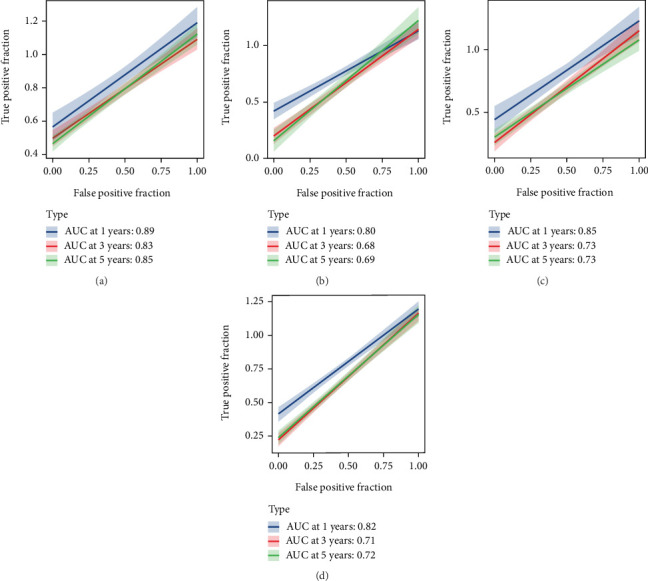
ROC analysis of the CoxBoost prognostic model across multiple cohorts. (a–d) ROC curves for the CoxBoost model in the TARGET (a), GSE16091 (b), GSE21257 (c), and GEO-total (d) cohorts. The AUC values remain between 0.7 and 0.8, indicating good predictive accuracy.

**Figure 5:**
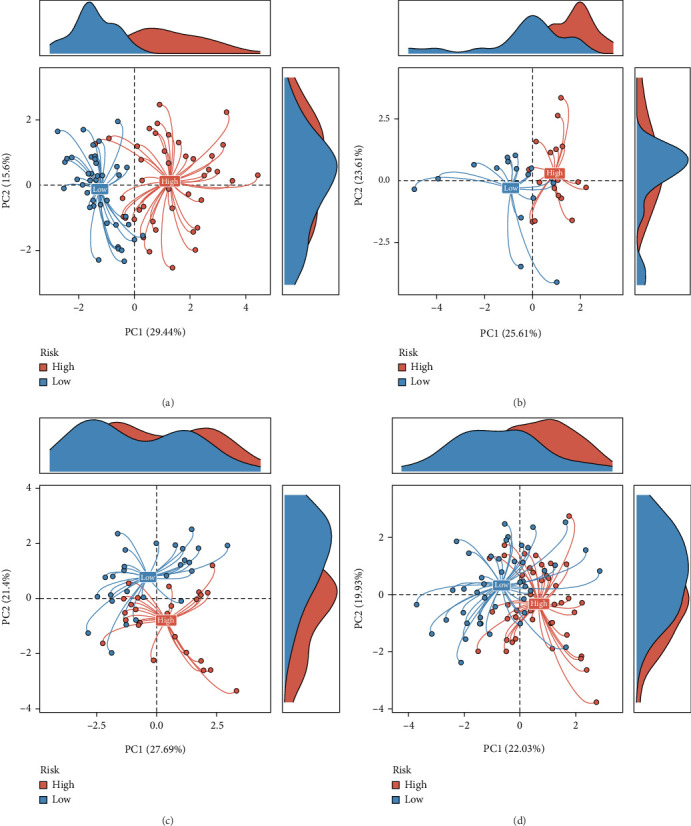
Principal component analysis (PCA) demonstrating prognostic model distinction. (a–d) PCA plots illustrating the separation of high-risk and low-risk patients within the TARGET (a), GSE16091 (b), GSE21257 (c), and GEO-total (d) cohorts. The model effectively distinguishes between different risk groups.

Using the CoxBoost prognostic model, we calculated risk scores in the TARGET cohort, GSE16091 cohort, GSE21257 cohort, and GEO-total cohort. Patients in each cohort were divided into high-risk and low-risk groups based on the median risk score. The results demonstrated that the high-risk group had significantly worse prognosis across all cohorts (Figures 3a, 3b, 3c, and 3d). The ROC curves showed that the AUC values for the prognostic model remained consistently between 0.7 and 0.8 across the four cohorts, indicating good model stability (Figures 4a, 4b, 4c, and 4d). PCA plots demonstrated that the prognostic model effectively distinguished patients within all four cohorts (Figures 5a, 5b, 5c, and 5d).

### 3.4. TME Analysis

**Figure 6:**
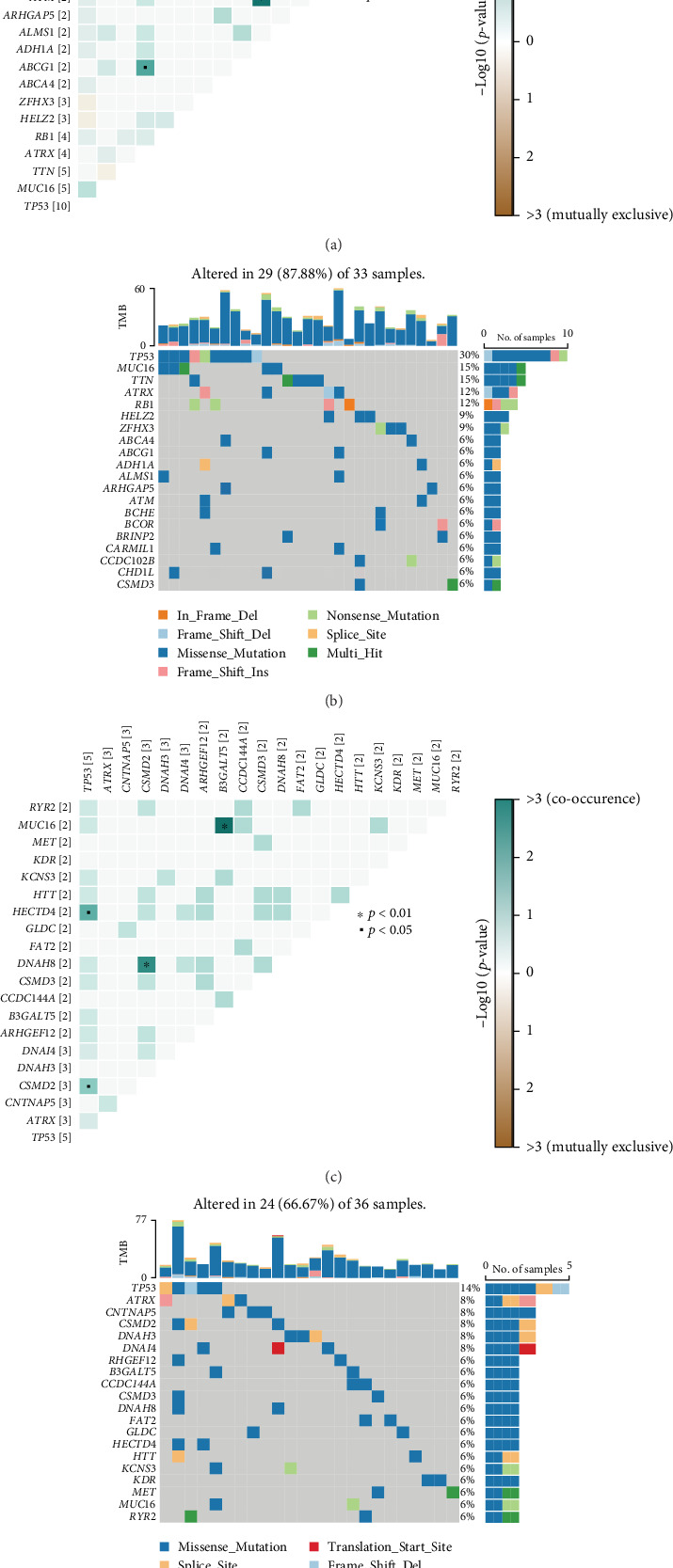
Mutation analysis of high-risk and low-risk groups in osteosarcoma. (a) Comutation network showing significant gene pair mutations in the high-risk group. (b) Heat map visualizing the mutation frequency of genes in the high-risk group. (c) Comutation network displaying significant gene pair mutations in the low-risk group. (d) Heat map illustrating the mutation frequency of genes in the low-risk group, with lower overall mutation frequency compared to the high-risk group.

First, gene mutation analysis was performed in the high-risk group, revealing notable comutations among MUC16 and CHD1L and ABCF1 and ATRX, as well as ATM and ADH1A (Figure 6a). Mutation frequencies in the high-risk group were further visualized in a heat map (Figure 6b). In the low-risk group, significant comutations were observed between HECTD4 and TP53, DNAH8 and CSMD2, and MUC16 and B3GALT5 (Figure 6c). The mutation frequencies were also plotted for the low-risk group, showing that the overall mutation frequency was lower compared to the high-risk group (Figure 6d).

**Figure 7:**
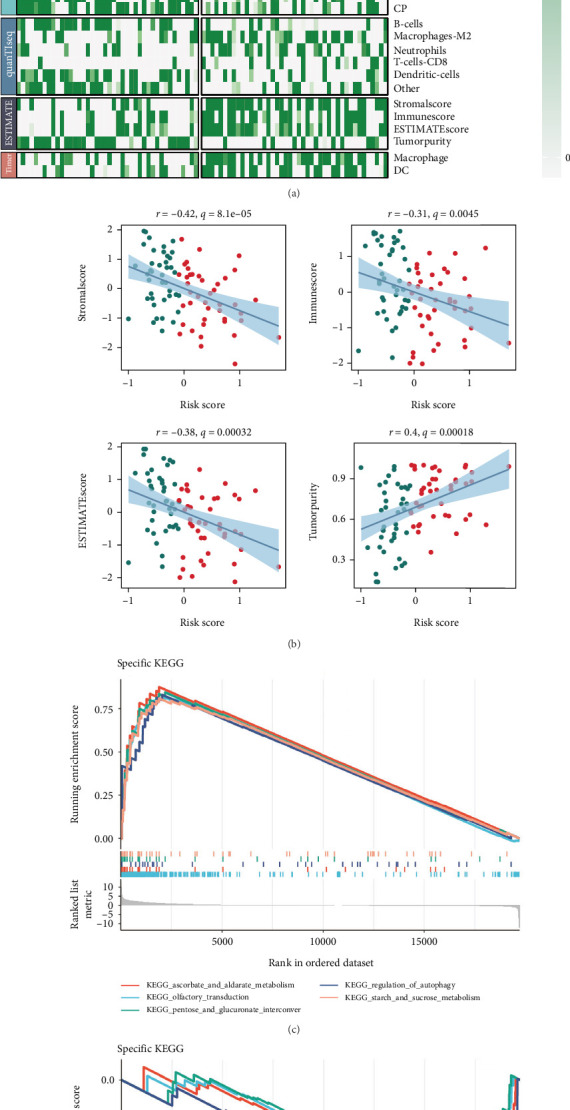
Tumor microenvironment and pathway analysis in high-risk and low-risk groups. (a) Heat map showing the differences in immune cell infiltration between high-risk and low-risk groups, with higher immune infiltration in the low-risk group. (b) Correlation analysis between risk score and stromal score, Immune score, ESTIMATE score, and tumor purity. Negative correlations are observed with stromal, immune, and ESTIMATE scores, while a positive correlation is seen with tumor purity. (c, d) GSEA plots highlighting differentially activated signaling pathways in the high-risk (c) and low-risk (d) groups.

Subsequently, we performed an in-depth immune cell infiltration analysis, which demonstrated that the low-risk group exhibited significantly higher levels of immune cell infiltration compared to the high-risk group (Figure 7a). This suggests that individuals within the low-risk group may possess a more robust immune response, potentially contributing to better prognosis and therapeutic responses. To further investigate the underlying relationships, we conducted a correlation analysis, which revealed a strikingly negative correlation between the risk score and both the stromal score, immune score, and ESTIMATE score, suggesting that a higher risk score is associated with reduced stromal and immune cell infiltration within the TME. In contrast, the risk score showed a positive correlation with tumor purity, indicating that tumors in the high-risk group may have a higher proportion of tumor cells relative to nontumor cells (Figure 7b).

To explore the molecular mechanisms behind these findings, GSEA was employed to compare the activation of biological pathways between the high-risk and low-risk groups. The analysis revealed distinct differences in pathway activations, with the low-risk group showing significant enrichment in immune-related pathways, highlighting a potentially enhanced immune response and favorable TME. On the other hand, the high-risk group demonstrated the activation of several pathways linked to tumor progression, metastasis, and immune evasion, underscoring the aggressiveness of these tumors (Figure 7c,d).

**Figure 8:**
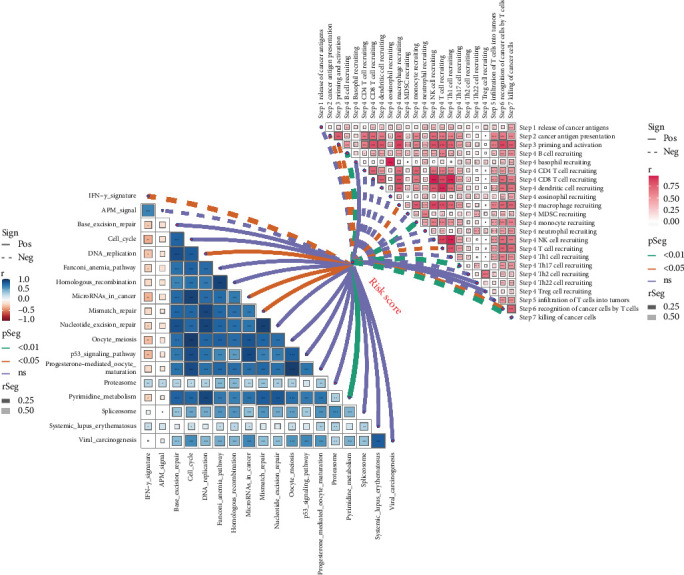
Correlation of risk score with immune cell recruitment and immune-related pathways.

Moreover, we observed that risk scores were strongly correlated with the recruitment of immune cells, including T cells, macrophages, and dendritic cells, all of which play critical roles in the antitumor immune response. The low-risk group, characterized by higher immune infiltration, may thus benefit from a more active immune surveillance (Figure 8).

**Figure 9:**
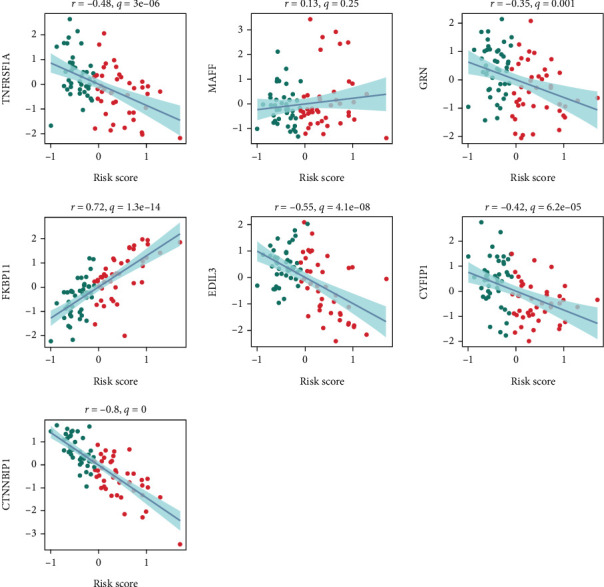
Validation of the prognostic model and FKBP11 expression in osteosarcoma.

As shown in Figure 9, the prognosis model was composed of seven genes, TNFRSF1A, MAFF, GRN, FKBP11, EDIL3, CYFIP1, and CTNNBIP1. FKBP11 is the most important positive-related gene. Collectively, these genes reflect diverse biological processes, including immune response, oxidative stress, angiogenesis, cytoskeletal reorganization, and signal transduction.

The prognostic model was constructed using seven genes: TNFRSF1A, MAFF, GRN, FKBP11, EDIL3, CYFIP1, and CTNNBIP1. Among these, FKBP11 was identified as the most significant positively correlated gene.

## 4. Discussion

OS is the most common primary malignant bone tumor, predominantly affecting children and adolescents [24]. Despite advancements in multimodal treatment strategies, including surgery, chemotherapy, and radiotherapy, the prognosis for patients with OS remains poor, particularly for those with metastatic or recurrent disease [25]. Understanding the complex TME and the various cell death pathways involved in OS is crucial for developing more effective therapeutic strategies [26]. In this context, PANoptosis, a unique inflammatory cell death pathway that integrates pyroptosis, apoptosis, and necroptosis, has recently garnered attention due to its potential role in tumor immunity and modulation of the TME [27–29].

This study provides a comprehensive analysis of the PANoptosis landscape in OS, revealing its heterogeneity across different cell types and highlighting its potential role in modulating the TME. The identification of distinct cell clusters with varying degrees of PANoptosis activation suggests that this inflammatory cell death pathway may contribute differentially to OS pathophysiology. Notably, CAFs, myeloid cells, and osteoblasts exhibited significant PANoptosis activation, implicating these cell types in the complex interplay between tumor cells and the TME.

The construction of a prognostic model using 101 machine learning algorithms represents a significant advancement in risk stratification for OS patients. Among these algorithms, the CoxBoost model demonstrated the most stable and consistent performance across multiple independent cohorts, underscoring its potential clinical utility. The model effectively distinguished between high-risk and low-risk patients, with the high-risk group consistently exhibiting worse survival outcomes. The robust performance of the model, as evidenced by the AUC values ranging from 0.7 to 0.8, further supports its potential as a reliable tool for guiding clinical decision-making.

The prognostic model identified in our study consists of seven genes: TNFRSF1A, MAFF, GRN, FKBP11, EDIL3, CYFIP1, and CTNNBIP1. Among these, FKBP11 emerged as the most significant positively correlated gene, indicating its strong association with adverse outcomes. FKBP11, a member of the FK506-binding protein family, has been implicated in various cellular processes, including protein folding and stress response, which may contribute to its role in tumor progression [30]. Similarly, TNFRSF1A and GRN are known to be involved in inflammatory and immune pathways [31], further suggesting their relevance to the TME and disease prognosis.

MAFF, a transcription factor responsive to oxidative stress, may play a role in regulating cellular adaptation mechanisms in tumors [32]. EDIL3, an extracellular matrix protein, has been linked to angiogenesis and metastasis, making it a key player in tumor invasion [33]. CYFIP1, a component of the WAVE regulatory complex, is associated with cytoskeletal dynamics and has been reported to act as a tumor suppressor in certain contexts [34]. Lastly, CTNNBIP1, a *β*-catenin interacting protein, is involved in the regulation of Wnt signaling, a pathway frequently dysregulated in cancer [35].

Collectively, these genes reflect diverse biological processes, including immune response, oxidative stress, angiogenesis, cytoskeletal reorganization, and signal transduction. Their inclusion in the model highlights the complex and multifactorial nature of tumor progression and provides potential targets for further mechanistic studies or therapeutic interventions.

Our findings also shed light on the tumor mutation landscape in OS, revealing distinct comutation patterns between high-risk and low-risk groups. The higher mutation frequency observed in the high-risk group may contribute to the aggressive nature of the disease, while the increased immune cell infiltration in the low-risk group suggests a more active immune surveillance, potentially leading to better outcomes. The significant correlations between risk scores and TME components, such as stromal and immune scores, highlight the intricate relationship between PANoptosis activation, the TME, and OS prognosis.

The limitations of this study include the reliance on publicly available datasets, which may introduce biases due to variations in data quality and experimental conditions. Additionally, while the machine learning–based prognostic model shows promise, further validation in prospective clinical trials is necessary to confirm its applicability in clinical settings.

In conclusion, this study provides valuable insights into the role of PANoptosis in OS and presents a novel prognostic model that may enhance patient stratification and inform therapeutic strategies. The integration of scRNA-seq data with machine learning approaches offers a powerful framework for unraveling the complexity of OS and advancing precision oncology in this challenging malignancy.

## Data Availability

The datasets analyzed in this study were downloaded and accessed from the Gene Expression Omnibus (GEO) database: https://www.ncbi.nlm.nih.gov/geo/, with Accession GSE162454.
